# Detection of codon 12 K- *ras* mutations in non-neoplastic mucosa from bronchial carina in patients with lung adenocarcinomas

**DOI:** 10.1054/bjoc.1999.0935

**Published:** 2000-01-18

**Authors:** T Urban, S Ricci, C Danel, M Antoine, M Kambouchner, V Godard, R Lacave, J-F Bernaudin

**Affiliations:** Department of Pneumology, Hôpital Saint-Antoine, 184 rue du Faubourg Saint-Antoine, Paris, 75012, France; Laboratory of Histology and Tumour Biology, Université Paris VI – Faculté de Médecine Saint-Antoine and Laboratory of Pathology, Hôpital Tenon, 4 rue de la Chine, Paris, 75020, France; Laboratory of Pathology, Hôpital Laennec, rue de Sèvres, Paris, 75006, France; Laboratory of Pathology, Hôpital Avicenne, 125 rue de Stalingrad, Bobigny, 95000, France

**Keywords:** ras oncogene, polymerase chain reaction, adenocarcinoma, bronchial mucosa

## Abstract

K- *ras* activation by point mutation in codon 12 has been reported in lung adenocarcinomas in various models of experimental lung tumours induced by chemical carcinogens. The hypothesis of the presence of cells containing K- *ras* mutation in non neoplastic bronchial carina, the main site of impaction of airborne contaminants, was investigated by evaluating concurrent lung tumour and non-neoplastic proximal bronchial carinae from 19 patients with lung adenocarcinomas. The restriction fragment length polymorphism enriched PCR method used can detect one mutant allele among 10^3^normal alleles. A mutation was detected in 42% of lung adenocarcinoma samples. No mutation was detected in either tumour or bronchial carinae in nine patients (47%). K- *ras* mutation was detected in the lung tumour but not in bronchial carinae in four patients (21%), in both the lung tumour and bronchial carinae in four other patients (21%). In two patients (11%), K- *ras* mutation was detected in at least one bronchial carina, but not in the lung tumour. Mutations of codon 12, confirmed by sequencing analysis of ten samples, were G to T transversion, mostly TGT and GTT in bronchial carinae and lung tumours. Our data show that activated K- *ras* by point mutation can be present in non-neoplastic bronchial carina mucosa even when no mutation is detected in tumour samples. © 2000 Cancer Research Campaign


*British Journal of Cancer* (2000) **82**(2), 412–417
© 2000 Cancer Research Campaign
Article no. bjoc.1999.0935

### Detection of codon 12 K-*ras* mutations in non-neoplastic mucosa from bronchial carina in patients with lung adenocarcinomas

**T Urban[1], S Ricci[2], C Danel[4], M Antoine[3], M Kambouchner[5], V Godard[2], R Lacave[2] and J-F Bernaudin[2]**

[1]Department of Pneumology, Hôpital Saint-Antoine, 184 rue du Faubourg Saint-Antoine, 75012 Paris, France; [2]Laboratory of Histology and Tumour Biology, Université Paris VI – Faculté de Médecine Saint-Antoine and [3]Laboratory of Pathology, Hôpital Tenon, 4 rue de la Chine, 75020 Paris, France; [4]Laboratory of Pathology, Hôpital Laennec, rue de Sèvres, 75006 Paris, France; [5]Laboratory of Pathology, Hôpital Avicenne, 125 rue de Stalingrad, 95000 Bobigny, France

**Summary** K-*ras* activation by point mutation in codon 12 has been reported in lung adenocarcinomas in various models of experimental lung tumours induced by chemical carcinogens. The hypothesis of the presence of cells containing K-*ras* mutation in non neoplastic bronchial carina, the main site of impaction of airborne contaminants, was investigated by evaluating concurrent lung tumour and non-neoplastic proximal bronchial carinae from 19 patients with lung adenocarcinomas. The restriction fragment length polymorphism enriched PCR method used can detect one mutant allele among $10^3$ normal alleles. A mutation was detected in 42% of lung adenocarcinoma samples. No mutation was detected in either tumour or bronchial carinae in nine patients (47%). K-*ras* mutation was detected in the lung tumour but not in bronchial carinae in four patients (21%), in both the lung tumour and bronchial carinae in four other patients (21%). In two patients (11%), K-*ras* mutation was detected in at least one bronchial carina, but not in the lung tumour. Mutations of codon 12, confirmed by sequencing analysis of ten samples, were G to T transversion, mostly TGT and GTT in bronchial carinae and lung tumours. Our data show that activated K-*ras* by point mutation can be present in non-neoplastic bronchial carina mucosa even when no mutation is detected in tumour samples.
© 2000 Cancer Research Campaign

**Keywords:** ras oncogene; polymerase chain reaction; adenocarcinoma; bronchial mucosa

The K-*ras* proto-oncogene is activated by point mutation in a wide variety of human and experimental carcinomas (Barbacid, 1987; Bos, 1989). Like others, we have reported that, in lung carcinomas, K-*ras* mutations were exclusively found in adenocarcinomas (Urban et al, 1996), with a frequency of 30% in smokers and 5% in non-smokers (Slebos et al, 1991), with more than 90% of K-*ras* mutations occurring in codon 12 (Barbacid, 1987; Slebos et al, 1991). In contrast, in another series, K-*ras* mutations were not only detected in adenocarcinomas, but also in squamous cell carcinomas (Rosell et al, 1993; Behn et al, 1998).

Experimental models of lung tumours conducted in various mouse strains with various chemical carcinogens (You et al 1989, 1993; Mass et al, 1993) and human data suggest that codon 12 of K-*ras* gene may be a target for the mutagenic activity of various tobacco smoke compounds (Sugio et al, 1992; Husafvel-Pursiaisen et al, 1993; Westra et al, 1993). K-*ras* mutation has been shown to be a preneoplastic event in colonic and probably pancreatic carcinogenesis (Loeb et al, 1984; Burmer and Loeis, 1989; Yanagisawa et al, 1993; Berthelemy et al, 1995; Brentall et al, 1995; Tada et al, 1996; Wilentz et al, 1998). Little is known about preneoplastic events in bronchopulmonary carcinogenesis, but K-*ras* and p53 mutations have been shown to occur early in the development of pulmonary adenocarcinoma (Sozzi et al, 1992; Sundaresen et al, 1992; Benett et al, 1993; Li et al, 1994). Loss of heterozygosity of 3p14 loci is also frequently observed in normal or metaplastic bronchial epithelium in smokers and ex-smokers (Mao et al, 1997).

The cellular targets for the carcinogenic compounds of tobacco smoke are usually considered to be either the bronchial mucosa or alveolar epithelium (Carney, 1991). In a previous study, testing the hypothesis of the presence of widespread target cells containing K-*ras* mutations in the respiratory tract, we did not find any mutation of codon 12 of the K-*ras* gene in non-neoplastic distal bronchial or parenchymal tissues in patients with lung adenocarcinoma (Urban et al, 1996). Similarly, in a recent report comparing neoplastic tissue and contralateral bronchial mucosa, the authors failed to detect K-*ras* mutations in non-neoplastic cells (Behn et al, 1998). In contrast, Clements et al (1995) reported K-*ras* mutations in non-malignant bronchial tissue in some patients with lung adenocarcinomas.

Bronchial carinae are the main sites of impaction of airborne contaminants such as tobacco smoke compounds (Knudson, 1960; Auerbach et al, 1979). It can be hypothesized that these areas may constitute targets for the genotoxic effects induced by carcinogenic compounds, such as K-*ras* gene mutations. As a preliminary step to test this hypothesis, we therefore investigated the occurrence of K-*ras* mutations in non-neoplastic bronchial carinae from a series of patients with lung adenocarcinomas with and without K-*ras* mutations. The study was performed on non-neoplastic bronchial carinae collected from lungs after thoracotomy for primary lung adenocarcinomas in 19 patients.

*Received 23 November 1998*
*Revised 30 March 1999*
*Accepted 29 June 1999*

*Correspondence to:* JF Bernaudin

**Table 1** Localization and presence of mutated K-*ras* gene (codon 12) in lung primary adenocarcinomas with their corresponding non-neoplastic carinae and the results of sequence of K-*ras* gene (codon 12) mutation

| | Lung adenocarcinomas | | | Non-neoplastic bronchial samples | | |
|---|---|---|---|---|---|---|
| | Localization | Codon 12 K-*ras* | | Localization (same side) | Codon 12 K-*ras* | |
| ***No.*** | | RFLP | Sequence | | RFLP | Sequence |
| Group 1 | | | | | | |
| 1 | Apical of apicodorsal (left upper lobe) | Mutated | GTT | Upper lobe division carina | Wild | – |
| 2 | Right upper bronchus | Mutated | GTT | Proximal carina | Wild | – |
| 3 | Peripheral in ventrobasal (left lower lobe) | Mutated | TGT | Lower segment carina | Wild | – |
| | | | | Lower lobe bronchus | Wild | – |
| | | | | Lower subsegmental carina | – | |
| 4 | Peripheral (right lower lobe) | Mutated | TGT | Segment carina | Wild | – |
| | | | | Subsegment carina | Wild | – |
| | | | | Subsegment carina | Wild | – |
| Group 2 | | | | | | |
| 5 | Peripheral (left lower lobe) | Mutated | – | Segment carina | Mutated | – |
| | | | | Segment carina | Mutated | – |
| 6 | Right upper lobar bronchus | Mutated | – | Upper lobe carina | Wild | – |
| | | | | Segment carina | Mutated | – |
| | | | | Segment carina | Mutated | – |
| | | | | Middle lobe carina | Wild | – |
| 7 | Peripheral (apical of left lower lobe) | Mutated | TGT | Segment carina | Wild | GGT |
| | | | | Segment carina | Mutated | TGT |
| | | | | Lower lobe bronchus section | Wild | GGT |
| 8 | Peripheral (apical of left upper lobe) | Mutated | TGT | Upper lobe bronchus | Wild | GGT |
| | | | | Segmental carina | Wild | GGT |
| | | | | Segmental carina (bis) | Mutated | TGT |

[a]Histologically normal; –, not done

### MATERIALS AND METHODS

### Tissue specimens

Lung specimens from 19 patients with lung adenocarcinomas were evaluated (Tables 1 and 2). All patients underwent preoperative staging and bronchoscopy with serial carinal biopsies for histological staging as a routine procedure. All but one (patient 13) of the patients were smokers or ex-smokers.

All tissue samples investigated in the present study were collected from specimens obtained after thoracotomy performed for curative intent with a curative resection, i.e. lobectomy or pneumonectomy. This study was therefore conducted in accordance with the rules of the Ethical Committee of our institution.

The resected lung was rapidly transported to the Pathology Department. After examination by the pathologist, non-neoplastic bronchial carina specimens away from the primary tumour, either from the same lobe in the case of lobectomy ($n$ = 14) or if possible from another lobe in the case of pneumonectomy ($n$ = 5), were collected and snap-frozen at – 70°C until analysis. The standard bronchial nomenclature was used to localize the tumour and carinae (Kitamura and Kobayashi, 1995). Only specimens from lungs containing a tumour situated away from the non-neoplastic carina were sampled. A representative part of the tumour was also snap-frozen and stored at –70°C until analysis. Tissue samples were embedded in paraffin wax for histological analysis and classified according to the WHO classification into adenocarcinomas. To increase the sensitivity of our method of detection of codon 12 K-*ras* mutations, macroscopically neoplastic tissue and bronchial carina were studied under a dissection microscope for DNA extraction.

### Control cell lines

HT29 (homozygous for the wild K-*ras* gene codon 12, glycine, GGT) and SW480 (homozygous for a mutated K-*ras* gene codon 12, valine, GTT) human colon carcinoma cell lines were obtained from the American Type Culture Collection (ATCC, Rockville, MD, USA). Tumour DNA with pre-determined codon 12 mutations identified by sequencing was also used as control DNA.

### Enriched PCR/RFLP analysis

K-*ras* codon 12 sequences were amplified (Perkin-Elmer Cetus thermal cycler) by an enriched polymerase chain reaction (PCR)–restriction fragment length polymorphism (RFLP) method, as previously reported (Kahn et al, 1991; Urban et al, 1996).

#### *First-step amplification*

The first PCR reaction used a K-*ras* 5′ primer (5′ ACT GAA TAT AAA CTT GTG GTA GTT GGA <u>C</u>CT 3′) containing a <u>C</u> substitution at the first position of codon 11 creating a *Bst*NI site which overlaps the first two nucleotides of codon 12, and K-*ras* 3′ wide-type (wt) primer (5 TCA AAG AAT GGT CCT GCA CC 3′) (part of exon 1 of K-*ras* gene). Fifteen cycles of a PCR reaction were performed with an annealing temperature of 56°C with the following reagents: 50 mM potassium chloride, 10 mM Tris–HCl pH 8.3, 1.5 mM magnesium chloride, 0.2 mM dNTPs, 2.5 units of *Taq* DNA polymerase (Boehringer Mannheim, France), and 10 ng each of K-*ras* 5′ and K-*ras* 3′ wt primers.

#### *Intermediate digestion*

Five-microlitre aliquots of the first PCR reaction were digested with 10 units of the restriction enzyme *Bst*NI (Boehringer

*© 2000 Cancer Research Campaign*

**Table 2** Localization of lung primary adenocarcinomas with wild-type K-*ras* gene (codon 12) with their corresponding non-neoplastic carinae and the results of mutated K-*ras* gene (codon 12) detection

| No. | Lung adenocarcinomas: Localization | Codon 12 K-*ras* RFLP | Codon 12 K-*ras* Sequence | Non-neoplastic bronchial carinae: Localization (same side) | Codon 12 K-*ras* RFLP | Codon 12 K-*ras* Sequence |
|---|---|---|---|---|---|---|
| Group 3 | | | | | | |
| 9 | Ventral of right upper lobe | Wild | GGT | Segment carina | Mutated | GTT |
| | | | | Segment carina | Wild | GGT |
| 10 | Apical of right lower lobe | Wild | GGT | Upper lobe carina | Mutated | – |
| | | | | Upper lobe carina | Mutated | TGT |
| | | | | Middle lobe carina | Wild | GGT |
| | | | | Lower lobe carina | Wild | GGT |
| Group 4 | | | | | | |
| 11a | Laterobasal of left lower lobe | Wild | GGT | Segment carina | Wild | GGT |
| | | | | Segment carina | Wild | GGT |
| 12 | Laterodorsobasal of left lower lobe | Wild | GGT | Segment carina | Wild | GGT |
| | | | | Segment carina | Wild | GGT |
| 13b | Left upper bronchus | Wild | – | Main bronchus | Wild | – |
| | | | | | Wild | – |
| 14 | Peripheral (right upper lobe) | Wild | GGT | Segment carina | Wild | – |
| 15 | Peripheral (right upper lobe) | Wild | GGT | Subsegmental carina | Wild | – |
| 16 | Peripheral (left upper lobe) | Wild | GGT | Upper lobe bronchus carina | Wild | – |
| 17 | Peripheral (right lower lobe) | Wild | GGT | Proximal lobe carina | Wild | – |
| 18 | Peripheral (dorsal of right upper lobe) | Wild | – | Upper lobe carina | Wild | – |
| | | | | Upper segment carina | Wild | – |
| | | | | Upper subsegment carina | Wild | – |
| 19 | Peripheral (apical of right upper lobe) | Wild | – | Upper lobe carina | Wild | GGT |
| | | | | Truncus intermedius | Wild | GGT |

[a]Adenosquamous lung carcinoma; [b]non-smoker patient; –, not done

Mannheim, France) in a final volume of 10 µl, at 37°C for 1 h under conditions recommended by the supplier.

### *Second-step amplification*

One microlitre of digested mixture was diluted to a final volume of 50 µl as described above. Primer concentrations were 150 ng each of K-*ras* 5′ (as described above) and K-*ras* 3′ primers (5′ TCA AAG AAT GGT CCT GGA CC3′), which also contain a substitution creating a control *Bst*NI site. Amplification was performed for 30 cycles, as described above. Thirty-microlitre aliquots obtained after this second step were snap-frozen at –30°C for further analysis.

### *RFLP analysis*

Twenty-microlitre aliquots of the PCR products obtained after the second step were digested with 10 units of the restriction enzyme *Bst*NI, at 37°C for 2 h in a final volume of 40 µl. The results were analysed by 8% polyacrylamide electrophoresis followed by ethidium bromide staining and UV transillumination. All specimens were re-evaluated in a second separate PCR amplification.

### Codon 12 sequences

Nucleotide sequences of ten mutated K-*ras* codon 12 detected after enriched PCR/RFLP analysis were determined by either a Genetic Analyser or specific oligonucleotide hybridization according to Hruban et al (1993). Twenty samples without K-*ras* mutation were also sequenced as controls.

Sequencing was performed with purified PCR/RFLP products obtained immediately after the second PCR amplification step (as described above) without second *Bst*NI reaction. Cycle sequencing was performed using the ABI PRISM™ dRhodamine Terminator Cycle Sequencing Ready Reaction Kit with AmpliTaq® DNA polymerase FS (Applied Biosystems, Paris) in a GeneAmp PCR systems 9600 (Perkin-Elmer). The precipitated pellets were resuspended in Template Suppression Reagent® (Applied Biosystems, Paris, France), denatured and electrophoresed on the ABI Prism 310 Genetic Analyser. The PCR product sequence was analysed with ABI PRISM™ software.

Oligonucleotide sequences, hybridization conditions in ammonium tetramethylchloride, specific washing conditions and composition of the probes used have been previously described by Wilentz et al (1998).

### RESULTS

### Patients

Nineteen patients with lung adenocarcinomas were studied. Non-neoplastic bronchial carina samples were available in all 19 patients, as reported in Tables 1 and 2. Surgical resection consisted of lobectomy ($n$ = 14) or pneumonectomy ($n$ = 5). Eight patients presented an endobronchial tumour diagnosed during a bronchoscopy procedure, whereas 11 patients had a peripheral lung tumour, mainly in a subpleural site. The tumours were classified according to the TNM classification as stage I (11/19), stage II (2/19), stage III (4/19) or stage IV (2/19). Two patients were classified as stage IV because of isolated cranial metastasis and underwent complete neurosurgical resection.

© 2000 Cancer Research Campaign

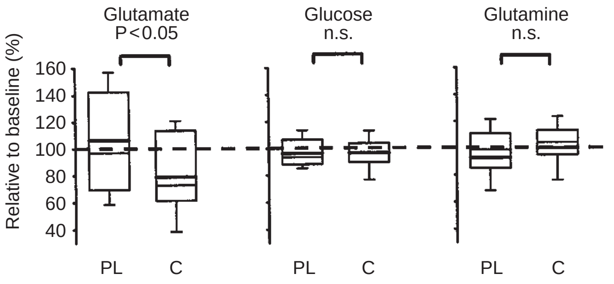

**Figure 1** Enriched PCR amplification and RFLP analysis. Photograph of DNA electrophoresis through an 8% polyacrylamide gel after ethidium bromide staining and UV analysis. Left and right lanes: molecular weight markers. The base pair number is indicated in the right margin. Lanes 1–4: Bst NI digestion of PCR product after amplification of DNA from three bronchial carina samples and lung adenocarcinoma sample with wild-type codon 12 showing a single 114 bp fragment. Lane 6: Bst NI digestion of PCR product after amplification of DNA from bronchial carina sample (patient no. 7) with wild-type codon 12 showing a single 114 bp fragment. Lane 5 and 7: BSTNI digestion of PCR product after amplification of DNA from samples from bronchial carina and lung adenocarcinoma (patient no. 7) with heterozygous mutation, showing 114 and 143 fragments. Lane 8: PCR product after amplification of DNA from lung adenocarcinoma (patient no. 7), before BstNI digestion, showing a single 157 bp fragment.

### Enriched PCR/RFLP method

SW480 and HT29 human colon carcinoma cell lines were used to validate our PCR/RFLP method. Amplification of the K-*ras* codon 12 sequence gave a 157 bp fragment before *Bst*NI digestion. After the two-step procedure described above, digestion of wild-type codon 12 sequence (HT29 cell line DNA) with *Bst*NI generated a 114 bp fragment, while, when a mutation was present in codon 12 (SW480 cell line DNA), *Bst*NI digestion generated a 143 bp fragment, as shown in Figure 1. The presence of a 143 bp fragment after digestion is therefore the hallmark of the presence of mutated K-*ras* genes on codon 12.

Analysis of a mixture of DNA from the two cell lines showed two fragments of 143 and 114 bp respectively. The sensitivity of the assay was therefore controlled by a series of titration experiments. The limit of detection was 1 cell with homozygous mutation within a minimum of $10^3$ cells with a wild-type K-*ras* gene, as previously reported (Urban et al, 1996).

### Detection of codon 12 c-K-*ras* mutations in patient samples

The detailed results are shown in Tables 1 and 2. A mutation in codon 12 of the K-*ras* gene was observed in eight of the 19 lung adenocarcinomas (42%) (Tables 1 and 2). A mutation in codon 12 of the K-*ras* gene was observed in nine of the 41 non neoplastic bronchial carina samples evaluated (22%), i.e. in six of the 19 patients (32%) (Tables 1 and 2).

### Lung tumour and non-neoplastic carinae

Four different patterns were observed, expressed as groups 1, 2, 3 and 4 (Tables 1 and 2).

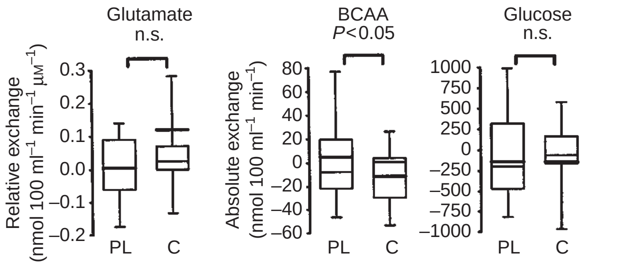

**Figure 2** Characterization of K-*ras* mutations by sequencing analysis. Shown is the sequence of codon 12 in 3 samples: **(A)** wild type K-*ras* (GGT); **(B)** mutated K-*ras* (GTT); **(C)** presence of wild-type (GGT) and mutated (TGT) K-*ras* alleles in the DNA sample

In group 1: (patients 1–4), a K-*ras* mutation was detected in lung tumours, but not in bronchial carinae (Table 1).

In group 2: (patients 5–8), K-*ras* mutations were detected in lung tumours and in bronchial carina samples collected away from the tumour. It must be stressed that, for patient 6, presenting a tumour located in the right upper lobe, no mutation was detected in the upper lobe carina, but a K-*ras* gene mutation was detected in the apical carina of the right lower lobe (Table 1).

In group 3: (patients 9 and 10), no mutation was detected in the tumours of these two patients, despite multiple ($n$ = 3) evaluation by enriched PCR/RFLP, but mutations were present in bronchial carinae (Table 2).

In group 4: (patients 11–19), no mutation was detected in either lung tumour or bronchial carina samples (Table 2).

### Codon 12 sequences

Detailed data are reported in Table 2. The type of codon 12 K-*ras* mutations was always G to T transversions, TGT and GTT, in both carina and lung tumours (Figure 2).

### DISCUSSION

The method used to detect mutation in the 12th codon of the K-*ras* gene was a combination of PCR DNA amplification and RFLP analysis (Jiang et al, 1989; Urban et al, 1993). This method is highly specific, as loss of the restriction site at the target is diagnostic for the presence of a mutation (Jiang et al, 1991; Urban et al, 1996; Behn et al, 1998). The enriched PCR/RFLP procedure is able to detect one mutated allele among $10^3$ normal alleles, while a one-step PCR/RFLP analysis is only able to detect a K-*ras* mutation present in 1% of cells studied (Urban et al, 1996). Used as a control of this method, specific oligonucleotide hybridization gave similar results in previously published experiments (Urban et al, 1996).

In the present series of 19 lung adenocarcinomas, activated protooncogene K-*ras* by mutation on codon 12 was observed in eight lung adenocarcinomas (42%), as already reported previously (Rodenhuis et al, 1988; Slebos et al, 1990; Urban et al, 1996). However, similar results have been recently reported; for example Ronai et al (1996) detected K-*ras* mutation in 48% of a series of non-small-cell lung carcinomas.

*© 2000 Cancer Research Campaign*

K-*ras* mutation has been shown to be a preneoplastic event in colonic and pancreatic carcinogenesis (Burmer and Loeb, 1989; Yanagisawa et al, 1993; Berthelemy et al, 1995; Brentall et al, 1995; Tada et al, 1996; Wilentz et al, 1998). Moreover, when using an enriched PCR method, K-*ras* mutations were detected in normal colonic mucosa away from the tumour (Minamoto et al, 1995). K-*ras* mutations have also been detected in mucous hyperplasia of pancreatic ducts associated with pancreatitis, suggesting that they may be precancerous epithelial changes in the pathogenesis of pancreatic carcinomas (Yanagisawa et al, 1993).

In contrast, data concerning preneoplastic events in bronchopulmonary carcinogenesis are more recent and incomplete. p53 mutations have been shown to occur very early in the development of lung adenocarcinomas (Sozzi et al, 1992; Sundaresen et al, 1992; Benett et al, 1993; Li et al, 1994), and a linkage with tobacco smoke exposure has been clearly established with the presence of genetic abnormalities in the p53 gene (Denissenko et al, 1996). In a recent study based on serial bronchial biopsies, Mao et al (1997) reported a high frequency of genetic abnormalities of the 3p14 loci (FHIT 3p14.2 gene) and, less frequently, of the 9p21 loci (p16 gene) and 17p13 loci (p53 gene) in normal and metaplastic bronchial epithelium sampled in smokers and non-smokers. Loss of heterozygosity of 3p14 chromosome was observed in 88% of smokers and 45% of ex-smokers, but was a very rare event in non-smokers, and was more frequently observed in the case of a high metaplastic index. This observation suggested a linkage with tobacco smoke exposure and potential reversibility of 3p14 genetic abnormalities in ex-smokers. In contrast, the early occurrence of K-*ras* mutation is still controversial as for some authors it is considered as a late event in the carcinogenesis of lung tumours (Sugio et al, 1994). However, our results as well as those previously reported by Clements et al (1995) suggest that K-*ras* mutation could be an earlier event than usually considered.

The cellular targets for the carcinogenic compounds of tobacco smoke are usually considered to be either the bronchial mucosa or alveolar epithelium (Carney, 1991). The hypothesis of the presence of widespread target cells containing K-*ras* mutations in the respiratory tract, as already shown for the affected suppressor gene p53, therefore had to be considered (Sozzi et al, 1992; Sundaresen et al, 1992; Benett et al, 1993; Li et al, 1994). We previously tested this hypothesis by evaluating distal tissues, i.e. non-proximal bronchial or parenchymal tissues, but failed to find any mutations on codon 12 of the K-*ras* gene in these tissues, in patients with lung cancers (Urban et al, 1996). In 1995, Clements et al reported K-*ras* mutations in non-malignant bronchial tissues collected under fibreoptic bronchoscopic examination in patients with non-small-cell lung carcinomas (Clements et al, 1995). We therefore hypothesized that such a discrepancy between these results (Li et al, 1994; Urban et al, 1996; Behn et al, 1998) could be explained by the fact that the samples studied by Clements et al were collected from proximal bronchial carinae, which are the main sites of impaction of tobacco smoke compounds, shown to be the site of epithelial changes in response to constant exposure to airborne contaminants (Knudson, 1960; Auerbach et al, 1979).

The present study shows that K-*ras* gene mutations in codon 12 can be present in non-neoplastic bronchial carina in patients with lung adenocarcinomas. These mutations were detected either away from a tumour harbouring a K-*ras* mutation, but also in two patients not presenting any mutation in the carcinoma, despite multiple evaluations by enriched PCR/RFLP, confirmed by sequencing analysis. This finding clearly demonstrates that the presence of mutated K-*ras*, at least in these three cases, was not related to a theoretical possibility of endobronchial contamination by tumour cells.

Codon 12 mutations were found to be G to T transversions, mostly TGT and GTT, in carina samples and lung tumours. These results are in accordance with previous studies (Kobayashi et al, 1990; Westra et al, 1993; Rodenhuis et al, 1997). Benzo(a)pyrene, and nitrosamines, considered to be the major carcinogenic compounds of tobacco smoke (Loeb et al, 1984; Carothers, 1990), have been demonstrated to induce mostly G to T transversion in experimental murine models of lung tumours harbouring a K-*ras* mutation restricted to codon 12 (You et al, 1989, 1993; Mass et al, 1993).

The significance of K-*ras* activation in lung carcinogenesis therefore remains to be elucidated. One hypothesis is that such a mutation could be a marker of exposure to tobacco smoke carcinogenic compounds in patients at risk for lung adenocarcinoma. In fact, in experimental murine lung tumour models performed in F1 littermates from susceptible and resistant parents, it has been shown that codon 12 mutations affect the K-*ras* allele from the susceptible parent (You et al, 1992). It has also recently been shown that K-*ras* polymorphism associated with two other polymorphic markers from chromosome 12p was associated with a risk of lung adenocarcinoma (Manenti et al, 1997).

In conclusion, mutated K-*ras* can be detected in non-neoplastic bronchial mucosa, particularly bronchial carina, which is the main site of impaction of airborne contaminants such as tobacco smoke compounds. The results of this study now need to be confirmed by a larger study conducted in smokers and non-smokers with or without various types of lung carcinomas.

### ACKNOWLEDGEMENTS

Prof. F Soubrier is gratefully acknowledged for his major help in sequencing assistance. V Gerber is gratefully acknowledged for secretarial assistance. This work was supported by grants from DRC AP-HP 98, ACTT 98, and crédits de l'Université Pierre et Marie Curie, 1998.

### REFERENCES

Auerbach O, Hammond EC and Garfinkel L (1979) Changes in bronchial epithelium in relation to cigarette smoking, 1955–1960 vs. 1970–1977. *N Engl J Med* **300**: 381–386

Barbacid M (1987) *ras* genes. *Annu Rev Biochem* **56**: 779–827

Behn M, Qun S, Pankow W, Havemann K and Schuermann M (1998) Frequent detection of ras and p53 mutations in brush cytology samples from lung cancer patients by a restriction fragment length polymorphism-based 'enriched PCR' technique. *Clin Cancer Res* **4**: 361–371

Benett WP, Colby TV, Travis WD, Borkowski A, Jones RT, Lane DP, Metcalf RA, Samet JM, Takeshima Y, GU JR, Vähäkangas KH, Soini Y, Pääkkoö P, Welsh JA, Trump BF and Harris CC (1993) p53 protein accumulates frequently in early bronchial neoplasia. *Cancer Res* **53**: 4817–4822

Berthelemy P, Bouisson M, Escourrov J, Vaysse N, Rumeau JL and Pradayrol L (1995) Identification of K-ras mutations in pancreatic juice in the early diagnosis of pancreatic cancer. *Ann Intern Med* **123**: 188–191

Bos JL (1989) ras oncogenes in human cancer: a review. *Cancer Res* **49**: 4682–4689

Brentnall TA, Chen R, Lee JG, Kimmey MB, Bronner MP, Haggitt RC, Kondley KV, Hecker LM and Byrd DR (1995) Microsatellite instability and K-ras mutations associated with pancreatic adenocarcinoma and pancreatitis. *Cancer Res* **55**: 4264–4267

Burmer GC and Loeb L (1989) Mutations in the K-ras oncogene during progressive stages of human colon carcinoma. *Proc Natl Acad Sci USA* **86**: 2403–2407

Carney DN (1991) Lung cancer biology. *Curr Opin Cancer* **3**: 288–296

© 2000 Cancer Research Campaign

Carothers AM and Grunberger D (1990) DNA base changes in benzo(a)pyrene diol epoxide-induced dihydrofolate reductase mutants of chinese hamster ovary cells. *Carcinogenesis* **11**: 189–192
Clements NC, Nelson MA, Wymer JA, Savage C, Aguirre M and Garewal H (1995) Analysis of K-ras gene mutations in malignant and non-malignant endobronchial tissue obtained by fiberoptic bronchoscopy. *Am J Respir Crit Care Med* **152**: 1374–1378
Denissenko MF, Pao A, Tang MS and Pfeifer GP (1996) Preferential formation of benzo(a)pyrene at lung cancer mutational hotspots in P53. *Science* **274**: 430–432
Hruban RH, Van Mansfeld ADM, Offerhaus GJA, Van Weering DH, Alison DC, Goodman SN, Kensler TW, Bose KK, Cameron JL and Bos JL (1993) K-ras oncogene activation in adenocarcinoma of the human pancreas. *Am J Pathol* **143**: 545–554
Husafvel-Pursiaisen K, Hackman P, Ridanpää M, Anttilas S, Karjalainen A, Tartanen T, Taikina-Aho O, Heikkila L and Vainio H (1993) K-ras mutations in human adenocarcinoma of the lung with smoking and occupational exposure to asbestos. *Int J Cancer* **53**: 250–256
Jiang W, Khan SM, Guillem JG, Lu SH and Weinstein B (1989) Rapid detection of ras oncogenes in human tumors: applications to colon, oesophageal, and gastric cancer. *Oncogene* **4**: 923–928
Kitamura S and Kobayashi J (1995) Atlas and basic technique. In: *Textbook of Bronchoscopy*, Feinsilver SH and Fein AM (eds), pp. 31–48. Williams and Wilkins: Baltimore
Khan SM, Jiang WJ, Culbertson TA, Weinstein B, Williams GM, Tomita N and Ronai Z (1991) Rapid and sensitive detection of mutant K-ras genes via ‘enriched’ PCR amplification. *Oncogene* **6**: 1079–1083
Knudson KP (1960) The pathologic effects of smoking tobacco in the trachea and bronchial mucosa. *Am J Clin Path* **33**: 310–317
Kobayashi T, Tsuda H, Noguchi M, Hirohashi S, Goya T and Hayata Y (1990) Association of point mutation in c-ki-ras oncogene in lung adenocarcinoma with particular reference to cytologic subtypes. *Cancer* **66**: 289–294
Leone-Klaber S, Wessner LL, McEntee MF, D’Agostino RB JR and Miller MS (1997) Ki-ras mutations are an early event and correlate with tumor stage in transplacentally-induced murine lung tumors. *Carcinogenesis* **18**: 1163–1168
Loeb LA, Ernester VL, Warner KE, Abbotts J and Lazlo J (1984) Smoking and lung cancer: an overview. *Cancer Res* **44**: 5940–5958
Li ZH, Zheng J, Weiss LM and Shibata D (1994) c-K-ras 2 and p53 mutations occur very early in adenocarcinoma of the lung. *Am J Pathol* **144**: 303–309
Manenti G, De Gregorio L, Pilotti S, Falvella FS, Incarbone M, Ravagnani F, Pierotti MA and Dragani TS (1997) Association of chromosome 12p genetic polymorphisms with lung adenocarcinoma risk and prognosis. *Carcinogenesis* **98**: 1917–1920
Mao L, Lee JS, Kurie JM, Kurie JM, Fan YH, Lippman SM, Lee JJ, RO JY, Broxson A, Yu R, Morice RC, Kemp BL, Khuri FR, Walsh GL, Hittelman WN and Hong WK (1997) Clonal genetic alterations in the lungs of current and former smokers. *J Natl Cancer Inst* **89**: 857–862
Mass MJ, Jeffers AJ, Ross JA, Nelson G, Galati AJ, Stoner GD and Nesnow S (1993) K-ras 2 oncogene mutations in tumors and DNA adducts formed by benz(j)aceanthrylene and benzo(a)pyrene in the lungs of strain A/J mice. *Mol Carcinogen* **8**: 186–192.
Minamoto T, Yamashita N, Ochiai A, Mai M, Sugimara T, Ronai Z and Esumi H (1995) Mutant K-ras in apparently normal mucosa of colorectal cancer patients. Its potential as a biomarker of colorectal tumorigenesis. *Cancer* **75**: 1520–1526
Rodenhuis S, Slebos RJC, Boot AJM, Evers SG, Mooi WJ, Wagenaar SSC, Van Bodegom PCh, and Bos JL (1988) Incidence and possible clinical significance of K-ras oncogene activation in adenocarcinoma of the human lung. *Cancer Res* **48**: 5738–5741
Rodenhuis S, Boerrigter L, Top B, Slebos RJ, Mooi WJ, Van’Tveer L, and van Zandwijk N (1997) Mutational activation of the K-ras oncogene and the effect of chemotherapy in advanced adenocarcinoma of the lung: a prospective study. *J Clin Oncol* **15**: 285–291
Ronai Z, Yabubovskaya MS, Zhang E and Belitsky GA (1996) K-ras mutation in sputum of patients with or without lung cancer. *J Cell Biochem* **25**: 172–176
Rosell R, Li S, Skacel Z, Mate JL, Maestre J, Canela M, Tolosa E, Armengol P, Barnabas A and Ariza A (1993) Prognosis impact of mutated K-ras gene in surgically resected non-small cell lung cancer patients. *Oncogene* **8**: 2407–2412
Slebos RJC, Kibbelaar RE, Dalesio O, Kooistra A, Stam J, Meijer CJ, Wagenaar SS, Vanderschueren RG, Van Zandwijk N, Mooi WJ, Bos JL and Rodenhuis S (1990) K-ras oncogene activation as a prognostic marker in adenocarcinoma of the lung. *N Engl J Med* **323**: 561–565
Slebos RJC, Hruban RH, Dalesio O, Mooi WJ, Offerhaus JA and Rodenhuis S (1991) Relationship between K-ras activation and smoking in adenocarcinoma of the human lung. *J Natl Cancer Inst* **83**: 1024–1027
Sozzi G, Miozzo M, Donghi R, Pilotti S, Cariani CT, Pastorino U, Della Porta G, and Pierotti MA (1992) Deletions of 17p and p53 mutations in preneoplastic lesions of the lung. *Cancer Res* **52**: 6079–6082
Sundaresan V, Ganly P, Hasleton P, Rudd R, Sinha G, Bleehen NM and Rabbitts P (1992) p53 and chromosome 3 abnormalities, characteristic of malignant lung tumours, are detectable in preinvasive lesions of the bronchus. *Oncogene* **7**: 1989–1997
Sugio K, Ishida T, Yokoyama H, Inoue T, Sugimachi K and Sasazuki (1992) Ras gene mutations as a prognostic marker in adenocarcinoma of the human lung without lymph node metastasis. *Cancer Res* **52**: 2903–2906
Tada M, Ohashi M, Shiratori Y, Okudaira T, Komatsu Y and Kawabe T (1996) Analysis of K-ras gene mutations in hyperplastic duct cells of the pancreas without pancreas disease. *Gastroenterology* **110**: 227–231
Urban T, Ricci S, Grange JD, Lacave R, Boudghene F, Breittmayer F, Languille O, Roland J and Bernaudin JF (1993) Detection of c-K-ras 2 mutations by PCR/RFLP analysis and diagnosis of pancreatic adenocarcinomas. *J Natl Cancer Inst* **85**: 2008–2012
Urban T, Ricci S, Lacave R, Antoine M, Kambouchner M, Capron F and Bernaudin JF (1996) Codon 12 ki-ras mutation in non-small cell lung cancer: comparative evaluation in tumoural and non-tumoural lung. *Br J Cancer* **74**: 1051–1055
Westra WH, Slebos RJC, Offerhaus GJA, Goodman SN, Evers SG, Kensler TW, Askin FB, Rodenhuis S and Hruban RH (1993) K-ras oncogene activation in lung adenocarcinomas from former smokers. *Cancer* **72**: 432–438
Wilentz RE, Chung C, Sturm PDJ, Musler A, Sohn TA, Offerhaus GJ, Yeo CJ and Hruban RH (1998) K-ras mutations in the duodenal fluid of patients with pancreatic carcinoma. *Cancer* **82**: 96–103
Yanagisawa A, Ohtake K, Ohashi K, Hori M, Kitagawa T, Sugano H and Kato Y (1993) Frequent c-K-ras oncogene activation in mucous cell hyperplasia of pancreas suffering from chronic inflammation *Cancer Res* **53**: 953–956
You M, Candrian U, Maronpot RR, Stoner GD, Marshall W and Anderson W (1989) Activation of the k-ras 2 proto-oncogene in spontaneously occurring and chemically induced lung tumors of the strain A mouse. *Proc Natl Acad Sci USA* **86**: 3070–3074
You M, Wang Y, Stoner G, You L, Maronpot R, Reynolds SH and Anderson M (1992) Parental bias of K-ras oncogenes detected in lung tumors from mouse hybrids. *Proc Natl Acad Sci USA* **89**: 5804–5808
You M, Wang Y, Nash B and Stoner GD (1993) K-ras mutations in benzotrochloride-induced lung tumors of A/J mice. *Carcinogenesis* **14**: 1247–1249.

*© 2000 Cancer Research Campaign* 
